# Novel case of resolution of hypsarrhythmia following tuber resection in a patient with infantile spasms and tuberous sclerosis

**DOI:** 10.1002/ccr3.780

**Published:** 2017-04-19

**Authors:** Robert Marsh, Courtney Nichols, Mary Payne

**Affiliations:** ^1^ Department of NeuroscienceMarshall University1600 Medical Center Drive, Suite B500Huntington25701West VirginiaUSA; ^2^ Present address: Department of NeurosurgeryWVU1 Medical Center Drive, Suite 4300Morgantown26506‐9183West VirginiaUSA

**Keywords:** Hypsarrhythmia, tuber resection, tuberous sclerosis

## Abstract

This article describes a case involving the resolution of hypsarrhythmia, a generalized abnormal EEG pattern, following focal resection of a cortical tuber in a patient with tuberous sclerosis.

Tuberous sclerosis is an autosomal‐dominant genetic disorder caused by a mutation in either the TSC1 gene on chromosome 9q34 or the TSC2 gene on chromosome 16p13 1. Tuberous sclerosis (TS) has a characteristic triad of clinical symptoms including various cutaneous manifestations, mental retardation, and epilepsy. More than 90% of patients diagnosed with tuberous sclerosis will initially present with seizures with more than 70% presenting within the first year of life, and more than 80% will have epilepsy 1, 2, 3. These seizures can vary in both frequency and severity and can range in character from focal seizures with or without impaired consciousness to generalized convulsive seizures. EEG findings in these patients often demonstrate focal spikes correlating with focal cortical tubers. When generalized patterns are recorded, such as generalized spike and wave activity, this is thought to be secondarily generalized from a focal epileptiform region.

Approximately one‐third of infants with TS will have infantile spasms 2. These infants may be diagnosed with West syndrome, which is defined by the triad of infantile spasms (IS), mental retardation, and a hypsarrhythmia pattern on EEG. The generation of hypsarrhythmia pattern in patients with tuberous sclerosis is not well understood 4. Patients diagnosed with both TS and West syndrome are known to have an overall poor prognosis including intractable seizures, abnormal EEG findings, developmental delays, lower IQs, and significant behavioral problems (including autism) 2, 5, 6, 7, 8. Even after infantile spasms resolve in these patients, studies have shown that all patients will progress to other forms of seizures, including both partial and generalized seizures 6.

Due to the various long‐term effects of intractable seizures in TS, it is important to attempt to control epilepsy at a young age. Most patients with TS have intractable seizures that are refractory to antiepileptic medications 1, 2. Other treatments options include a ketogenic diet, vagal nerve stimulator, or surgery 9. Surgical treatment for seizures in tuberous sclerosis has been shown to improve seizure frequency and long‐term outcomes 1, 2, 8, 9, 10, 11. Baumgartner et al. described four patients, three of which had IS, and all underwent focal resection with dramatic improvement in seizure frequency. In this study, age at presentation ranged from 5 months to 5 years with surgical resection occurring between 5 years and adulthood. In these patients, focal resections were targeted in conjunction with focal interictal spikes. Guerreiro et al. described 12 patients with TS and intractable seizures who all showed improvement in seizure frequency and severity following focal surgical resection of epileptogenic lesions that correlated well by imaging, EEG, and clinical symptoms 1.

In this report, we present the case of a 13‐month‐old girl with tuberous sclerosis who presented with a large right frontal cortical tuber and hypsarrhythmia on EEG. EEG also showed multifocal spikes, with the majority occurring in the right frontal region. Seizures consisted of a tonic spasm with electrographic right frontal onset. Following tuber resection, interictal multifocal spikes remained and right focal spikes were still prominent. However, the EEG hypsarrhythmic pattern resolved and seizure frequency decreased.

Our patient was initially diagnosed with tuberous sclerosis at birth following detection of a cardiac rhabdomyosarcoma. At that time, an EEG was performed to detect any abnormalities and found multifocal spikes. The patient began having seizures soon afterward that were initially described as staring spells, but progressed to generalized convulsive seizures. She was started on phenobarbital, which led to apparent worsening of her seizures. The patient then presented to our institution for a second opinion at 4 months of age. Levetiracetam was added at that time with only minor benefit. An MRI of the brain revealed a large right frontal cortical tuber with mass effect on the left frontal region (see MRI 1 Fig. 1). A repeat EEG carried out at 6 months of age showed hypsarrhythmia with multifocal spikes most frequent in the right frontal region (see EEG 1). Background activity consisted of high‐amplitude activity and chaotic waveforms. Seizure semiology varied, but the most consistent seizure types included complex partial seizures with staring, secondarily generalized seizures with generalized tonic clonic activity, and tonic spasms. EEG captured a tonic spasm, and its electrographic correlate consisted of right frontal low‐amplitude rhythmic spikes with rapid spread of electrographic activity to the left frontal region, then electrographic spread to the adjacent temporal and central regions. Ultimately, secondary generalization occurred (EEG 2). Vigabatrin was initiated, and the dose was increased to 150 mg/kg/day.

**Figure 1:**
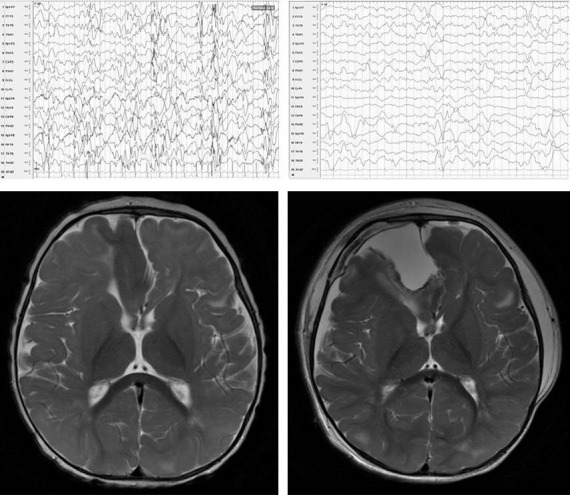
Upper left: hypsarrhythmia with predominant frontal spikes, lateralized to the right. Upper right: Absence of hypsarrhythmia with right frontal slowing and right frontal sharp waves. Lower left: MRI brain showing presence of large right frontal cortical tuber. Lower right: MRI brain following tuber removal.

Initially, seizure frequency and severity improved, and the EEG background was lower in amplitude with a regional differentiation and organization. However, this improvement was transient. Thus, it was decided to resect the frontal tuber. The patient's frontal tuber was resected. Pathology revealed brain tissue containing balloon cells with eosinophilic cytoplasm and dystrophic calcifications, consistent with a cortical tuber. Repeat EEG after surgery to remove the frontal tuber revealed resolution of hypsarrhythmia and appearance of regional differentiation. Another EEG was performed 7 months after surgery and again demonstrated absence of hypsarrhythmia and presence of regional organization.

In this case report, we have presented a unique case of an infant with tuberous sclerosis and West syndrome who had resolution of her interictal hypsarrhythmia pattern on EEG following surgical resection of a right frontal cortical tuber. Although other reports have described seizure improvement and even infantile spasm suppression with focal cortical tuber resection in either focal or generalized epileptiform discharges, this novel case describes resolution of hypsarrhythmia with tuber resection 7. Removal of a focal cortical tuber in an area of glucose hypometabolism seen on PET scan in patients with TS and hypsarrhythmia has previously been shown to improve seizure control and consequently resulted in improved neurological and behavioral development, but no EEG following surgery was shown to have demonstrated resolution of the hypsarrhythmia 4. A previously reported literature review of focal cortical resection in 177 patients with TS and intractable seizures showed complete resolution of seizures in 75%, with seizure onset ranging from age 1 day to 11 years and surgical resection performed between 3 months and 54 years of age 9. This review also looked at corpus callosotomy for multiple patients, showing improvement in seizure frequency, but no cases of seizure cessation following corpus callosotomy 9.

Even though cortical tuber resection has been shown to decrease seizure frequency, several previous studies have questioned where the cortical tubers characteristic of TS is epileptogenic, or whether these tubers induce surrounding cortex to become epileptogenic are themselves electrically silent. Some focal cortical tubers have been shown on ECoG to be electrographically silent, while the brain cortex surrounding these tubers was the origin of epileptiform activity 3. Other focal cortical tubers have been shown on intraoperative ECoG to possess epileptiform discharges intrinsically 1. While studies still debate the precise origins of epileptiform activity in TS, these studies have still all supported the premise that surgical resection of the focal cortical tubers results in improved seizure control and enhanced developmental improvement 1, 3, 4, 9.

Surgical intervention for intractable seizures in TS is primarily indicated in patients where an epileptogenic abnormality can be correlated with a single cortical tuber, imaging abnormalities, EEG findings, and clinical symptoms. Overall prognosis is also greatest in patients with little to no development or behavioral delays at time of surgery. Surgical intervention is also indicated in patients with intractable seizures whose EEG shows a pattern of secondary generalized epilepsy 1, 11.

While infantile spasms resolve over time, all patients with TS have been shown to progress to other forms of seizures following resolution of IS 5, 6. These seizures may include tonic spasms or complex partial seizures with EEG demonstrating generalized discharges, focal spikes, or both 2, 7. The type of epilepsy that develops following IS resolution may indicate the long‐term outcome for these patients, with generalized epilepsy having a worse prognosis than localized epilepsy 6.

A long‐term study of the intellectual capacity of TS patients following focal surgical tuber resection showed an objective improvement in patient's developmental age, social skills, and significant improvement in control of both seizure frequency and severity 11. By improving seizure control earlier in patients diagnosed with TS and West syndrome through focal surgical resection, we may be able to minimize developmental delays, improve social skills and behavioral issues, and avoid loss of cognitive function in these patients. In addition, patients with hypsarrhythmia often have poor long‐term outcomes in regard to seizure control and cognition. Thus, the decision to perform tuber resection should be multifactorial to include not only seizure control, but also preservation of cognitive functioning. We present a novel case of resolution of hypsarrhythmia following tuber resection, which may offer benefit to future individual patient outcome.

## Authorship

RM: is the neurosurgeon who resected frontal cortical tuber. He also contributed to writing the manuscript. CN: is a medical student who compiled the information, images, constructed the article, and performed the literature search. MP: is the neurologist who treated the patient's seizures, performed the EEG, and managed medical decision making.

## Conflict of Interest

None declared.
